# Aging of blood can be tracked by DNA methylation changes at just three CpG sites

**DOI:** 10.1186/gb-2014-15-2-r24

**Published:** 2014-02-03

**Authors:** Carola Ingrid Weidner, Qiong Lin, Carmen Maike Koch, Lewin Eisele, Fabian Beier, Patrick Ziegler, Dirk Olaf Bauerschlag, Karl-Heinz Jöckel, Raimund Erbel, Thomas Walter Mühleisen, Martin Zenke, Tim Henrik Brümmendorf, Wolfgang Wagner

**Affiliations:** 1 Helmholtz-Institute for Biomedical Engineering; Stem Cell Biology and Cellular Engineering, RWTH Aachen University Medical School, Aachen, Germany; 2 Institute for Biomedical Engineering - Cell Biology, RWTH Aachen University Medical School, Aachen, Germany; 3 Institute for Medical Informatics, Biometry and Epidemiology, University Duisburg-Essen, Essen, Germany; 4 Department of Oncology, Hematology and Stem Cell Transplantation, RWTH Aachen University Medical School, Aachen, Germany; 5 Department of Obstetrics and Gynecology, RWTH Aachen University Medical School, Aachen, Germany; 6 Department of Cardiology, West-German Heart Center Essen, University Duisburg-Essen, Essen, Germany; 7 Institute of Human Genetics, University of Bonn, Bonn, Germany; 8 Department of Genomics, Life and Brain Center, University of Bonn, Bonn, Germany; 9 Institute of Neuroscience and Medicine (INM-1), Research Center Juelich, Juelich, Germany

## Abstract

**Background:**

Human aging is associated with DNA methylation changes at specific sites in the genome. These epigenetic modifications may be used to track donor age for forensic analysis or to estimate biological age.

**Results:**

We perform a comprehensive analysis of methylation profiles to narrow down 102 age-related CpG sites in blood. We demonstrate that most of these age-associated methylation changes are reversed in induced pluripotent stem cells (iPSCs). Methylation levels at three age-related CpGs - located in the genes *ITGA2B*, *ASPA* and *PDE4C* - were subsequently analyzed by bisulfite pyrosequencing of 151 blood samples. This epigenetic aging signature facilitates age predictions with a mean absolute deviation from chronological age of less than 5 years. This precision is higher than age predictions based on telomere length. Variation of age predictions correlates moderately with clinical and lifestyle parameters supporting the notion that age-associated methylation changes are associated more with biological age than with chronological age. Furthermore, patients with acquired aplastic anemia or dyskeratosis congenita - two diseases associated with progressive bone marrow failure and severe telomere attrition - are predicted to be prematurely aged.

**Conclusions:**

Our epigenetic aging signature provides a simple biomarker to estimate the state of aging in blood. Age-associated DNA methylation changes are counteracted in iPSCs. On the other hand, over-estimation of chronological age in bone marrow failure syndromes is indicative for exhaustion of the hematopoietic cell pool. Thus, epigenetic changes upon aging seem to reflect biological aging of blood.

## Background

Aging reflects accumulation of cellular changes, due to either stochastic defects or a regulated developmental process [1]. This process is usually measured chronologically, although it does not perfectly correlate with time: 'biological age' is influenced by additional parameters such as genetic background, disease and lifestyle. Biomarkers for biological aging are relevant for geriatric assessment and may support the adaptation of habits to assist healthy aging [2]. Leukocyte telomere length has been suggested as a marker for biological age [3]. In fact, telomere attrition seems to be enhanced by various parameters, such as obesity and cigarette smoking [4]. Several other molecular methods can be used to estimate human age, including analysis of age-dependent deletions of mitochondrial DNA [5] or T-cell DNA rearrangements [6], and protein alterations such as racemization of aspartic acid [7] and advanced glycation end products [8]. However, all of these biomarkers have relatively low precision and practical limitations [9].

The epigenetic landscape provides new perspectives for biomarkers. In particular, DNA methylation (DNAm) is well known to change during aging [10]. Various recent studies have demonstrated the presence of age-related CpG sites (AR-CpGs), which are either hypermethylated or hypomethylated [11-14]. These DNAm changes are significantly enriched in bivalent chromatin domain promoters [15] and Polycomb group protein target genes [16-18], indicating that they might be governed by a developmental process. It is still not clear how epigenetic modifications are regulated during aging or if they rather reflect an increased deviation of local DNAm levels due to loss of control at specific loci [19]. Some CpG sites reveal almost linear DNAm changes during aging and can therefore be used for age prediction [19-21]. Bocklandt *et al.*[20] described a predictor of age in saliva samples generated using DNAm profiles from 34 twin pairs, indicating that age prediction based on just a few CpG sites is feasible, although this has not been validated in an independent set of samples. More recently, Hannum *et al.*[19] built a quantitative model of aging using DNAm values of 71 CpG sites that has been validated on various independent datasets. However, these studies were based on Illumina BeadChip technology, a method that requires complex bioinformatic analysis. In this study, we address the question of whether an assay based on bisulfite pyrosequencing of just a few CpG sites might be a less costly, faster, and user-friendly approach with similar accuracy to DNAm profiling approaches. To this end, we pooled publicly available DNAm profiles derived from blood samples to identify AR-CpG sites that, when combined, best predict donor age. Based on this analysis we developed an epigenetic aging signature that requires measurement of DNAm levels at only three CpG sites by bisulfite sequencing to facilitate reliable age predictions. Notably, these age predictions are influenced by clinical and lifestyle parameters, indicating that they are more indicative of biological age than chronological age.

## Results

### Age-related DNAm changes in blood samples

**Figure 1:**
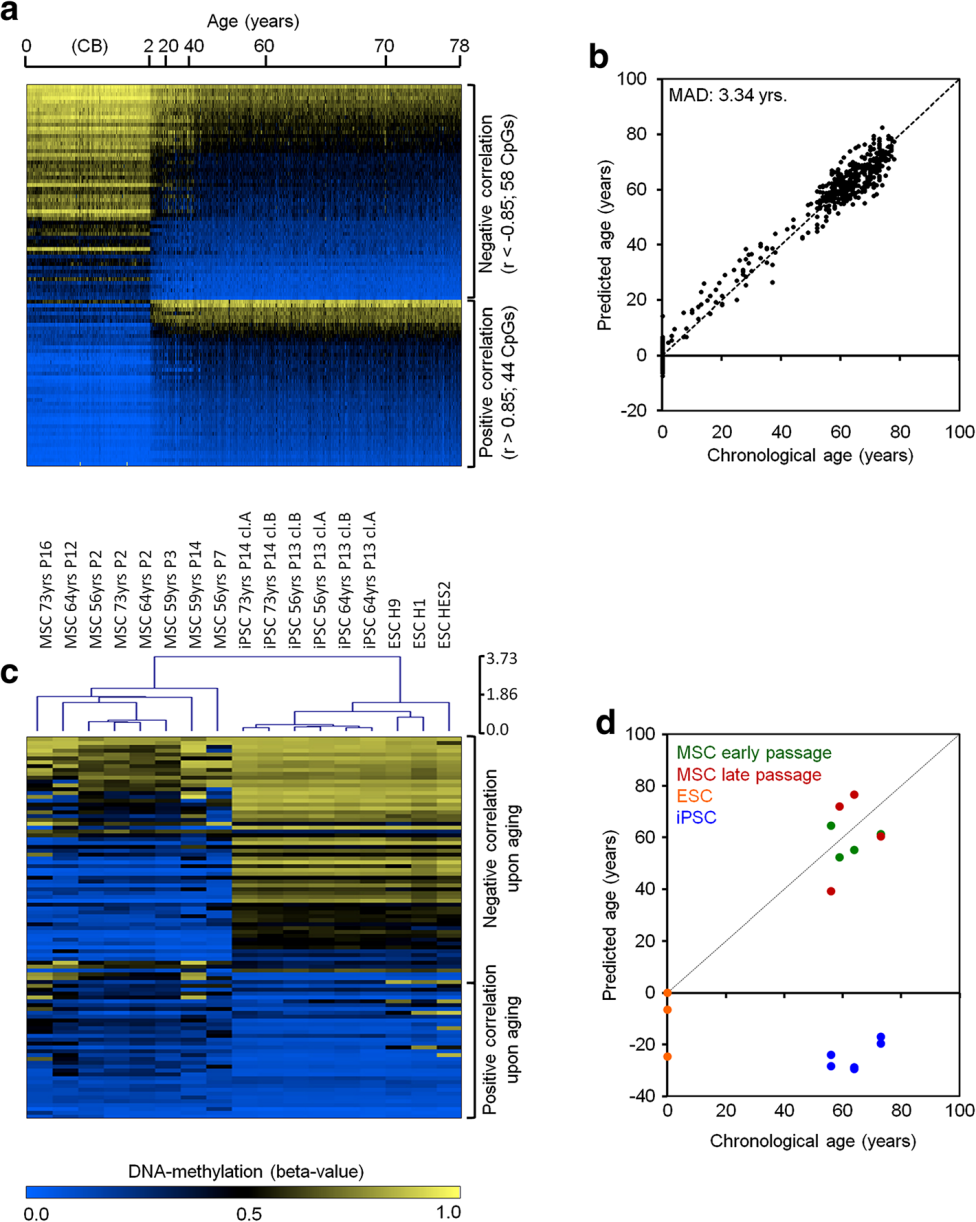
**Age-associated DNAm is reversed by reprogramming into induced pluripotent stem cells. (a)** A heatmap of 102 AR-CpG sites from 575 DNAm profiles derived from blood cells from donors of different ages (HumanMethylation27 BeadChip platform). All of these loci revealed relatively linear DNAm changes during aging (r < −0.85 or r > 0.85). **(b)** Based on these AR-CpGs, we generated a multivariate model to predict donor age and these predictions were compared to the corresponding chronological age. A combination of all 102 AR-CpGs facilitated reliable age predictions with a mean absolute deviation (MAD) of about 3.34 years. **(c)** Age-related-CpGs were subsequently analyzed in mesenchymal stromal cells (MSCs), induced pluripotent stem cells (iPSCs), and embryonic stem cells (ESCs) (heatmap clustered by Euclidean distance). Overall, AR-CpGs that are hypomethylated during aging are highly methylated in pluripotent stem cells and *vice versa*. **(d)** Subsequently, we used a multivariate model to predict donor age in these cells (early passage, P2 or P3; late passage, P7 to P16). Notably, iPSCs generated from these MSCs as well as ESCs were predicted to be of negative age, indicating that AR-DNAm changes are, overall, reversed by reprogramming into pluripotent cells.

We combined 575 DNAm profiles derived from blood cells from four different studies spanning an age range of 0 to 78 years (Table S1 in Additional file 1) [15,16,22,23]. All of these DNAm profiles were generated with the HumanMethylation27 BeadChip platform, which covers 27,578 individual CpG sites [24]. AR-CpGs with linear DNAm changes during aging were selected by Pearson correlation (either r > 0.85 or r < −0.85): 102 CpG sites passed these stringent parameters, including 58 hypomethylated and 44 hypermethylated CpGs (Figure 1a; Additional file 2).

In particular, the hypomethylated AR-CpGs were associated with genes involved in 'hematopoietic regulation', indicating that these age-related DNAm (AR-DNAm) changes reveal some tissue specificity (Table S3 in Additional file 1). Hypermethylated AR-CpGs were enriched within GC-rich sequences (Figure S1a in Additional file 1) [13]. Predicted transcription factor binding sites within 1 kb up- and downstream of AR-CpG sites differed considerably for hypo- and hypermethylated loci (Figure S1b in Additional file 1). Furthermore, only hypermethylated CpGs were significantly enriched in regions with bivalent histone modifications in embryonic stem cells (ESCs) and the H3K27me3 (trimethylation of lysine 27 of histone 3) marker in monocytes and mononuclear cells (Figure S2 in Additional file 1) [25,26], which has also been described before [15]. Overall, age-associated hypermethylation occurred particularly in regions with low DNAm levels, whereas age-related hypomethylation occurred at highly methylated regions. This trend towards a moderate methylation level has recently been described by other authors and may support the notion that many AR-DNAm changes are due to epigenetic drift evoked by increasing entropy of CpG markers, which tends towards 50% (Figure S3 in Additional file 1) [19,27]. Either way, the underlying mechanism resulting in AR-DNAm changes at specific genomic regions seems to differ for hypo- and hyper-methylated CpGs.

We trained a multivariate linear model to predict donor age based on the 102 AR-CpG sites selected by Pearson correlation. The results correlated well with chronological age with a mean absolute deviation (MAD) from chronological age of only 3.34 years (root mean square error (RMSE) = 4.26 years; R^2^ = 0.98; Figure 1b). These AR-CpG sites were further validated in three other publicly available datasets derived from blood samples [28-30] using the same multivariate linear model: the MAD from chronological age in these datasets was only slightly higher (GSE49904, 5.79 years; GSE41037, 5.52 years; GSE37008, 4.02 years; Figure S4a in Additional file 1). Furthermore, we considered the recently published dataset by Hannum and co-workers [19] of 656 DNAm profiles derived from blood samples (from donors aged 19 to 101 years). This dataset has been analyzed on the HumanMethylation450 BeadChip, which assays 485,577 CpG sites, including 99 of the 102 AR-CpG sites [31]. When we applied our multivariate linear model to this dataset, there was a clear correlation between age prediction and chronological age (R^2^ = 0.71). However, the linear offset indicated a systematic bias that might be due to the three missing CpG sites or to the different assay design of the two microarray platforms [31]. Therefore, we adjusted the multivariate regression model to facilitate age predictions based on these 99 AR-CpG sites, similar to the above-mentioned model (MAD, 4.12 years; RMSE, 5.34 years; R^2^ = 0.87; Figure S4b,c in Additional file 1).

### Age-related DNAm changes are counteracted in pluripotent stem cells

We have recently demonstrated that senescence-associated DNAm changes - which accumulate during long-term culture of cells *in vitro* - can be reversed by reprogramming into induced pluripotent stem cells (iPSCs) [32,33]. Here, we analyzed if AR-DNAm changes are also reversed in this dataset upon reprogramming: although our aging model had been trained on freshly isolated blood samples, it enabled moderate estimations of age in culture-expanded mesenchymal stromal cells as well. Notably, AR-DNAm changes were hardly affected by replicative senescence during culture expansion *in vitro*, indicating that AR-DNAm changes are not identical to DNAm changes acquired during *in vitro* culture (Figure 1c). Interestingly, CpG sites that are hypermethylated during aging are hypomethylated in pluripotent cells and *vice versa*. Using our multivariate model, the ESCs and iPSCs were even predicted to be of negative age (Figure 1d), which may reflect the reversal beyond the new-born state to the embryonic cell state. However, when we applied our multivariate model to a dataset with 19 undifferentiated human ESC lines and 5 iPSC lines (GSE34869) [34], they were predicted to have a mean donor age of −0.06 and 5.20 years, respectively (Figure S5 in Additional file 1). Thus, the deviation of ESCs and especially of iPSCs from zero might also be due to culture conditions or the comparison between different datasets. Either way, the data clearly indicate that AR-DNAm changes are, overall, reversed upon reprogramming. These findings fit nicely with other recent observations that iPSCs generated from senescent cells or centenarian donors reset telomere length, gene expression profiles, and other physiological features to those of young cells [35,36]. In addition, our results indicate that iPSCs are rejuvenated also on the epigenetic level.

### Selection of the epigenetic aging signature

**Figure 2:**
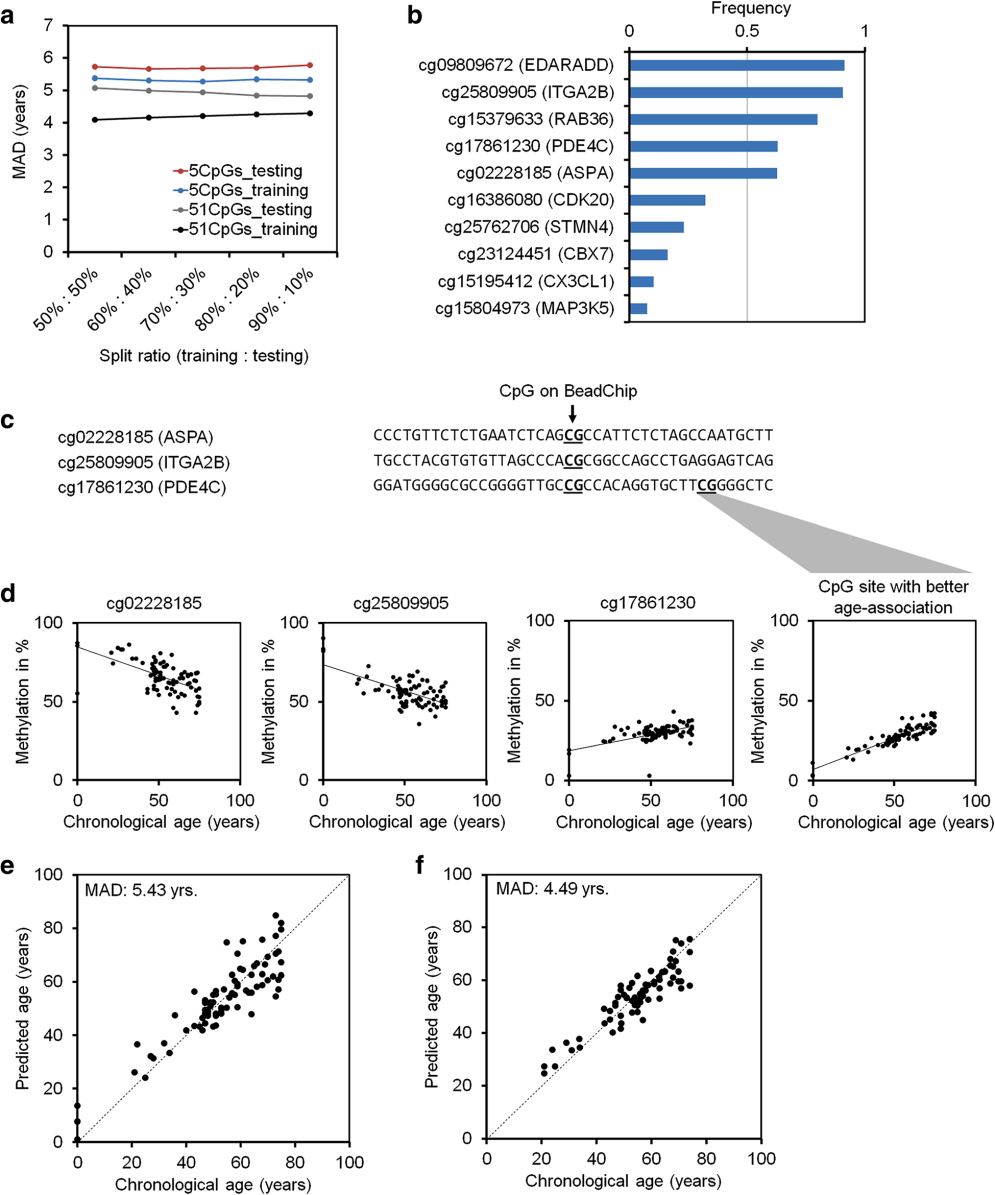
**Development of the epigenetic aging signature. (a)** The most relevant AR-CpGs were selected by iterative division of 575 DNAm profiles into training and testing sets (different split ratios). Age predictions were made for training sets using either 51 AR-CpGs or subsets of 5 CpGs. The results indicated that subsets with five CpGs (selected by recursive feature elimination) can enable age predictions with a mean absolute deviation (MAD) from chronological age of less than 6 years. **(b)** The frequency of occurrence of individual AR-CpGs in the best performing subsets of five CpGs. Five specific CpGs occurred in more than 50% of these filtered subsets and hence seemed to provide the best complement for age predictions. **(c)** DNAm at relevant AR-CpG sites was subsequently analyzed by pyrosequencing after bisulfite conversion. The sequences surrounding three of the five AR-CpGs were particularly suitable for this approach (CpG sites represented on the HumanMethylation27 BeadChip platform are indicated). **(d)** DNAm levels at these AR-CpGs were analyzed in a training set from 82 blood samples. The results were in line with the microarray data and revealed a clear age-associated correlation for each of the three CpGs. For cg17861230 (*PDE4C*) this correlation was even better at a neighboring CpG locus, which was therefore preferred for further analysis. **(e)** Based on the results with these three AR-CpGs, we generated a multivariate model that enabled relatively precise age predictions (MAD of 5.4 years). **(f)** Notably, the precision was even slightly higher when we validated this method in an independent set of 69 samples (MAD of 4.5 years).

Analysis of DNAm in a small subset of AR-CpGs might be sufficient for robust age predictions. Restriction to the most relevant CpGs would facilitate site-specific analysis of DNAm instead of profiling approaches. Therefore, we searched for subsets of AR-CpGs that, when combined, yield the best age predictions. AR-CpGs with the highest variation in DNAm levels were used for recursive feature elimination to select subsets of five CpG sites for testing in multivariate linear regression models. Predictions for each of these subsets were made by iterative divisions of the dataset into training and test sets (split ratios; Figure 2a). For further analysis we considered only those subsets of five CpGs that performed better than the average of models based on all 51 AR-CpG sites (Figure S6 in Additional file 1). Five specific CpGs occurred in more than 50% of the remaining subsets, indicating that they complement each other for age prediction (Figure 2b). These CpG sites are associated with EDAR-associated death domain (*EDARADD*), which has been associated with AR-DNAm changes before [20]; integrin, alpha 2b (*ITGA2B*); RAB36, a member of the RAS oncogene family (*RAB36*); phosphodiesterase 4C, cAMP specific (*PDE4C*); and aspartoacylase (*ASPA*) (Figure S7 in Additional file 1). Nevertheless, gene expression profiles indicated that expression of the five corresponding genes is hardly affected by aging (Figure S8 in Additional file 1) [37].

DNAm at these CpG sites was subsequently analyzed by pyrosequencing after bisulfite conversion in an independent training set derived from 82 blood samples. The sequences in the vicinity of cg09809672 (*EDARADD*) and cg15379633 (*RAB36*) were not ideal for the primer design and therefore we focused on the remaining three AR-CpGs. As expected, we observed a clear age-associated correlation for each of the three CpGs. In fact, the upstream located CpG site cg17861230 (*PDE4C*), which was covered by the same pyrosequencing assay, revealed an even better age-association and was therefore considered instead (Figure 2c,d). A multivariate linear regression model based on these pyrosequencing results facilitated age predictions with a MAD from chronological age of 5.4 years (RMSE, 7.2 years; Figure 2e). The corresponding equation is provided in the Material and methods section. To further simplify application of the epigenetic aging signature, we have compiled an online calculator that implements this equation [38].

Subsequently, we used this method on an independent validation set derived from 69 blood samples; this validation set was analyzed two months after the training set. DNAm levels at the three relevant CpGs were integrated into the above mentioned linear regression model established with the training set. Notably, the predictions for the validation set correlated even better with chronological age (MAD, 4.5 years; RMSE, 5.6 years; Figure 2f), indicating that pyrosequencing of these three CpG sites enables reliable age prediction.

### AR-DNAm changes are not due to changes in the cellular composition of blood

Our epigenetic age predictions might also be influenced by differences in the cellular composition in blood that result from aging [30]. Between the second and seventh decade a moderate decline in lymphocytes [39] and erythrocytes [40] has been described. When we analyzed DNAm of the three relevant CpG sites (cg02228185 in *ASPA*, cg25809905 in *ITGA2B*, and cg17861230 in *PDE4C*) in a publicly available dataset of cell type-specific DNAm profiles [41] the results indicated that the age predictions made using our epigenetic aging signature were not evoked by myeloid skewing (Figure S9a in Additional file 1). Furthermore, we made age predictions based on the three AR-CpGs using publicly available DNAm profiles of fractionated monocytes (CD14), T cells (CD4), granulocytes (CD16), and hematopoietic stem and progenitor cells (CD34) (GSE20242; E-MTAB-487) [15,42]. The results demonstrated that age predictions were feasible in purified cell populations, even though the MAD from chronological age was higher (Figure S9b in Additional file 1). Alternatively, we used another dataset to determine if the percentage of monocytes, lymphocytes, neutrophils, basophils, or eosinophils correlates with predicted age and there were no clear associations (GSE37008; Figure S10 in Additional file 1) [30]. These results indicate that AR-DNAm changes are due to intrinsic DNAm changes rather than to changes in cellular composition.

### Clinical and lifestyle parameters

**Figure 3:**
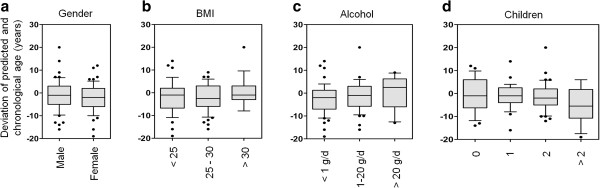
**Parameters with age-independent impacts on AR-CpGs.** Age predictions with our epigenetic aging signature were associated with various clinical and lifestyle parameters (105 samples from the HNR study). Deviations from chronological age revealed a moderate association with **(a)** gender (*P* = 0.28), **(b)** body mass index (BMI; *P* = 0.67), **(c)** alcohol consumption (*P* = 0.049), and **(d)** number of children (*P* = 0.0043 for females).

In an exploratory analysis to determine whether deviation of predicted age and chronological age correlated with other co-variables - such as clinical or lifestyle parameters - we focused on the 105 samples from the population-based prospective Heinz Nixdorf Recall (HNR) study [43]. Generally, age was estimated to be higher in men and in obese people (body mass index >30). These trends are in line with previous studies demonstrating an association with telomere length [44-46], but our results were not significant, which may be explained by the relatively small sample size (Figure S11 in Additional file 1). High alcohol consumption was also associated with overestimation of age (0.091 ± 0.045 years deviation per gram alcohol consumed per day; *P* = 0.049). Notably, age was increasingly underestimated according to increasing number of children in women (categories, 0, 1, 2, ≥3 children; -2.52 ± 0.84 years deviation per category; *P* = 0.0043), which has been associated with a longer lifespan before (Figure 3) [47].

### Age predictions correlate with telomere length

**Figure 4:**
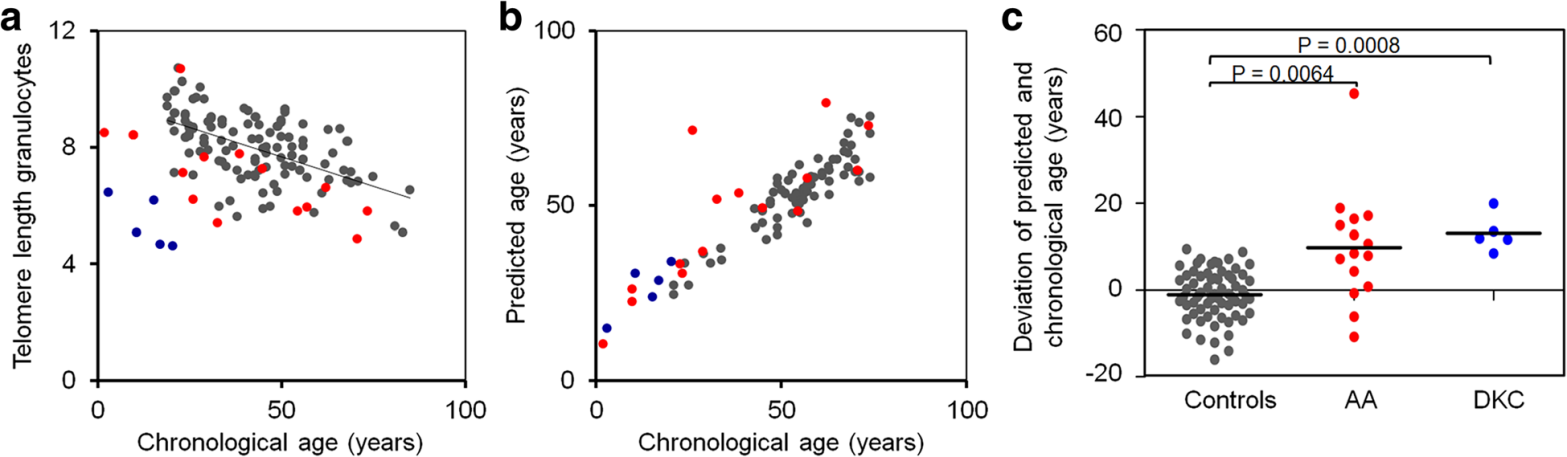
**Age-related DNAm correlates with telomere length. (a)** Telomere length of granulocytes was analyzed by flow-FISH in samples from 104 healthy donors (grey), patients with aplastic anemia (AA; red) and dyskeratosis congenita (DKC; blue). **(b, c)** Age predictions with our epigenetic aging signature demonstrated that several patients with AA or DKC - particularly those with telomere attrition - were predicted to be significantly older than their chronological age (in comparison to age predictions for the validation set in Figure 2f).

Telomere length is well known to decline during aging - an average of 39 bp per year in granulocytes [48] - and this approach can also be used to estimate donor age. We analyzed telomere length in 104 blood samples (from donors aged 18 to 84 years) by flow-FISH. Despite a clear inverse correlation between telomere length and chronological age, the precision of age predictions based on telomere length was relatively low (MAD, 18.2 years; RMSE, 23.1 years; Figure 4a; Figure S12a in Additional file 1). Extremely shortened telomeres have been reported in severe acquired aplastic anemia (AA) and patients with dyskeratosis congenita (DKC), both of which are associated with progressive bone marrow failure syndromes. Telomere attrition in these patients might result either from increased hematopoietic cell turnover due to autoimmune-mediated depletion of the hematopoietic stem cell pool, or, particularly in DKC, due to direct functional impairment of the telomerase complex by inactivating mutations [49,50]. We analyzed blood from 15 AA and 5 DKC patients, which revealed significantly shorter telomeres than healthy controls (Figure S12b in Additional file 1). Notably, these samples were predicted to be significantly older than their chronological age using our epigenetic aging signature, which may reflect replicative exhaustion of the hematopoietic stem cell pool (Figure 4b,c) [18,49].

## Discussion

We describe a method to predict donor age using blood samples based on DNAm at three specific CpG sites. The model is based on locus-specific pyrosequencing analysis of bisulfite-converted DNA, an approach that is relatively cost effective and does not require complicated bioinformatics. Most importantly, the precision of our method is much better than that of alternative non-epigenetic approaches such as measurement of telomere length, DNA rearrangements or protein alterations [9]. This assay can be used for forensic analysis of blood samples - theoretically, it can even be scaled down for relatively small traces of blood. Furthermore, our method may enable estimation of biological age using blood. Such predictions may be useful for geriatric assessment and may help tailor lifestyle to improve the odds of staying healthy. We provide evidence that deviation of predicted age and chronological age might be associated with specific lifestyle parameters - for example, gender, body mass index, alcohol consumption and the number of children - but most of these effects were not significant, which might be attributed to the relatively small sample size. On the other hand, aging may vary between tissues and therefore risk factors for age-related diseases - for example, in the cardiovascular system - are not necessarily reflected in the hematopoietic system.

AR-DNAm changes were suggested to be a tissue-specific phenomenon [17,51]. Yet, we and other authors have demonstrated that similar - but not identical - DNAm changes are acquired in different tissues [15,16,19,21]. The epigenetic aging signature described here has been specifically designed for use with blood, which is advantageous for practical diagnostics. Isolation of specific subsets, such as B cells, T cells, or neutrophils, would be difficult to implement in daily routine, even if it would further increase the precision of the method. Another advantage is that DNA is relatively stable and can be shipped at room temperature. Notably, the method even enabled age predictions using samples that had been cryopreserved for more than 10 years.

It is still not clear if and how AR-DNAm changes are controlled. Our data support the notion that epigenetic drift - in terms of Shannon entropy - occurs in certain regions that trend towards a DNAm level of 50% [19]. This may indicate that AR-DNAm changes are due to loss of DNAm maintenance at specific regions; hypermethylation happens in regions associated with repressive histone marks [15] and polycomb group protein targets [16], whereas hypomethylation may be enriched with other histone modifications, transcription factor binding sites or chromatin organizations. Whether AR-DNAm changes are the cause or consequence of aging also remains to be elucidated. Even though many of these AR-DNAm changes, including of the CpGs from our epigenetic aging signature, are not reflected in gene expression levels, it is well conceivable that the global epigenetic shift that occurs during aging entails loss of function in the elderly [52].

On the other hand, we provide evidence that AR-DNAm changes can be reversed by reprogramming into iPSCs. We have previously described that senescence-associated DNAm changes, which are acquired during long-term culture of mesenchymal stromal cells *in vitro*, are counteracted in iPSCs [33]. We have also demonstrated that AR-DNAm changes and senescence-associated DNAm changes differ considerably despite significant overlap [14]. This is in line with findings of this study as 102 AR-CpGs could not separate mesenchymal stromal cells into those of early and late passage. The observation that AR-DNAm changes - as well as senescence-associated DNAm changes - are reset to ground state supports the notion that pluripotency resets the aging clock: iPSCs generated from senescent cells and cells from centenarian donors have restored telomere length and their gene expression and cellular physiology appear to be indistinguishable from those of ESCs [35,36]. Notably, these cells can give rise to new organisms that - at the beginning - do not reveal any signs of aging. It is still not clear whether this 'epigenetic rejuvenation' can be disengaged from developmental reprogramming or whether it is inherently associated with passing through the pluripotent state [53]. The observation that AR-DNAm changes can be reversed indicates that, molecularly, their acquisition over a lifetime might be avoided and this is compatible with the evolutionary theories of aging [54]. In this regard, aging would not resemble inevitable accumulation of genetic aberrations, but rather a continuous deviation from the epigenetic ground state - a loss of control that is not counteracted in somatic cells and might be species- and tissue-specific.

Bone marrow failure in acquired AA and DKC has been suggested to be associated with extensive proliferation of hematopoietic stem cells [49,55]. DKC resembles an hereditary syndrome evoked by mutations in the telomerase complex or telomere-associated proteins whereas AA is acquired due to autoimmune processes, toxic compounds, or unknown factors. The degree of telomere shortening in AA is correlated with disease duration, stage and severity as well as with response to disease-modifying treatment strategies [49]. In this regard, it is striking that those samples with stark telomere attrition in particular are also predicted to be prematurely aged by our epigenetic aging signature. This observation further substantiates our method, which may provide a diagnostic measure for the replicative exhaustion of the hematopoietic stem cell pool.

## Conclusions

Our comprehensive analysis of DNAm profiles discerned AR-CpGs in blood. DNAm at three of these CpGs - determined, for example, by bisulfite pyrosequencing - can be used as a biomarker to enable age predictions with a MAD from chronological age of about 5 years. The notion that bone marrow failure syndromes, which are associated with telomere attrition, reveal premature aging also at the epigenetic level may indicate that AR-CpGs depict exhaustion of the hematopoietic stem cell pool. Although our exploratory analysis suggests some association with clinical or lifestyle parameters, it is still not known if this analysis reflects biological age of the organism or rather of the hematopoietic system. It is also not yet clear how AR-DNAm changes, which seem to occur in a coordinated and reversible manner, are governed and if they are functionally relevant. AR-DNAm changes may impact on chromatin structure or non-coding RNAs even without having an immediate impact on gene expression. Our epigenetic aging signature provides a simple approach that can be used to track the aging process, which may be useful when further trying to detail the underlying molecular mechanisms.

## Materials and methods

### Blood samples

We used blood samples from the HNR study, which is a prospective population-based cohort study (105 samples) [43,56] from the Department of Obstetrics and Gynecology of the University Hospital Aachen (GYN; 27 samples), and from the Department of Oncology, Hematology and Stem Cell Transplantation of the University Hospital Aachen (HEM; 104 samples from healthy donors, 15 AA, 5 DKC). All samples were taken after written consent and according to the guidelines of the local ethics committees.

### DNAm profiles and selection of AR-CpGs

We considered all DNAm profiles derived from blood samples that were generated with the HumanMethylation27 BeadChip platform and available at the time (Table S1 in Additional file 1). Beta values were combined and 102 AR-CpG sites were selected by Pearson correlation (r > 0.85 or r < −0.85). We trained a multivariate linear model for these AR-CpGs and applied leave-one-out cross-validation to estimate the model's performance. Age association of these CpGs was then tested using datasets GSE49904 [28], GSE41037 [29], GSE37008 [30], GSE34869 [34], and GSE20242 [15], and E-MTAB-487 [42]. We also used dataset GSE40279 [19], although this was generated with the HumanMethylation450 BeadChip (different assay design with type II bead type and only 99 of 102 AR-CpGs) [31] and we therefore trained an alternative model for use with this platform.

### Bioinformatics

Nucleotide sequences around each AR-CpG were retrieved from the human NCBI36/hg18 assembly. The frequency of nucleotides in the flanking regions was determined for 10 up- and downstream positions. FIMO (Find Individual Motif Occurrences) from the MEME Suite was utilized to scan for known transcription factor binding motifs within 1 kb flanking each CpG sites. The five most significantly enriched motifs in relation to all CpG sites on the array were depicted (*P*-values were estimated by Fisher’s exact test). The sequence logo plots were generated by the R seqLogo packages. Histone modifications at AR-CpG loci were analyzed using chromatin immunoprecipitation data for ESCs [25] (GSE8463 [26]; GSE29611, ENCODE project), CD14^+^ monocytes (GSE29611, ENCODE project), and MNCs (GSE31755, ENCODE project). Enrichment of H3K4me3, H3K27me3, the bivalent state or neither was estimated in relation to all CpG sites (Fisher’s exact test). Gene Ontology classification of genes associated with AR-CpGs was performed with GoMiner software [57] in relation to all CpGs on the BeadChip (Fisher’s exact test). For the five most important AR-CpGs, we analyzed gene expression data from the Leiden Longevity Study (150 samples; GSE16717) [37], which were generated with the 54 k CodeLink Human Whole Genome Bioarray. Methylation profiles of our top three AR-CpGs in subsets of different blood cells were analyzed using another dataset (GSE35069) [41].

### Selection of CpGs for the epigenetic aging signature

To obtain a small set of AR-CpGs for age predictions based on locus-specific DNAm levels, we narrowed 102 AR-CpGs down to those with above median variations in beta values (above median level of interquartile ranges). The dataset was then randomly divided into training and validation sets by defined split ratios. Next we applied recursive feature elimination implemented in the R caret package [58] for a limited feature size of five CpGs using a linear model on the training set. Relevant CpG sites were then selected by their frequency in the best performing models (Figure S6 in Additional file 1). The five most relevant AR-CpGs were subsequently considered for locus-specific DNAm analysis by pyrosequencing. The sequences surrounding cg02228185 (*ASPA*), cg25809905 (*ITGA2B*), and cg17861230 (*PDE4C*) were best suited for this approach.

### Pyrosequencing and age predictions

Genomic DNA was isolated from GYN samples with the QIAamp DNA Blood Midi Kit (QIAGEN, Hilden, Germany; GYN), from HEM samples with the DNeasy Mini Kit (QIAGEN), and from the HNR study samples using the Chemagic Magnetic Separation Module I (Chemagen, Baesweiler, Germany). Subsequently, 500 ng DNA were bisulfite-converted using the EpiTect Bisulfite Kit (QIAGEN). Converted DNA was amplified and 12 μl (*ASPA, ITGA2B*) or 20 to 25 μl (*PDE4C*) of PCR product was immobilized to 2 μl Streptavidin Sepharose™ HP beads (GE Healthcare, Piscataway, NJ, USA) followed by annealing to 1.0 μl sequencing primer (5 μM) for 2 minutes at 80°C. Primers for pyrosequencing analysis are listed in Table S4 in Additional file 1. Analysis was performed with PyroMark Q24 software. Initially, pyrosequencing was performed for 82 blood samples (27 GYN and 55 HNR) at Varionostic GmbH (Ulm, Germany); these samples were entirely independent from the DNAm profiles that were used to identify the three genomic locations. Based on the pyrosequencing results of the initial 82 blood samples, we generated the multivariate model. Beta values at the following three CpGs were used for age-prediction: (α) = cg02228185; (β) = cg25809905, and (γ) = a CpG site upstream of cg17861230 that revealed better correlation with age (Figure 2c).

$$\mathrm{Predicted}\;\mathrm{age}\;\left(\mathrm{in}\;\mathrm{years}\right)=38.0‒26.4\;\alpha ‒23.7\;\beta +164.7\;\gamma$$

This equation resembles also the underlying source code for the freely available online calculator [38]. This simple model was subsequently validated with an independent set of 69 samples (19 HEM and 50 HNR).

### Biostatistics on clinical parameters

To estimate the impact of co-variables (for example, lifestyle or clinical parameters), we calculated univariate linear regression models using the deviation of predicted age and chronological age as dependent variable and the respective co-variable as independent variable (SAS, version 9.2, Cary, New Jersey, USA). Similar models were computed separately for males and females to assess gender differences. Some 52 male and 53 female donors from different age categories (intervals of 6 years per age category) were selected randomly from the HNR study (4,814 participants) [56]. Box-and-whisker plots (10 to 90 percentiles) were plotted with GraphPad Prism 5 (GraphPad Software, La Jolla, CA, USA). Statistical significance was assessed from statistical significance of parameter estimates of the linear regression model depicting the change in deviation per one unit increase in the corresponding co-variable.

### Analysis of telomere length and age prediction

Telomere length of granulocytes and lymphocytes was analyzed in 104 samples from healthy donors aged 18 to 84 years, 15 patients with AA, and 5 with DKC (all HEM) by flow-FISH as described before [59,60]. In brief, samples were analyzed in triplicates with and without FITC-(C3TA2)_3_ PNA or Alexa488-(C3TA2) PNA (for healthy controls or AA and DKC, respectively; Panagene, Daejeon, South Korea). Cow thymocytes with known telomere length were used as an internal control to calculate telomere length in kilobases. The cow thymocytes as well as granulocytes and lymphocytes from human samples were identified based on forward scatter and LDS 751 binding to double-stranded DNA. For flow-FISH, telomere length was determined in absolute values. Age-related telomere length was estimated by linear regression on the 104 blood samples from healthy donors.

## Abbreviations

AA: aplastic anemia; AR-CpG: age-related CpG site; AR-DNAm: age-related DNA methylation; bp: base pair; DKC: dyskeratosis congenita; DNAm: DNA methylation; ESC: embryonic stem cell; FISH: fluorescent in situ hybridization; HNR: Heinz Nixdorf Recall; iPSC: induced pluripotent stem cell; MAD: mean absolute deviation; RMSE: root mean square error.

## Competing interests

The authors declare that they have no competing interests. RWTH Aachen University has applied for a patent for the epigenetic aging signature.

## Authors' contributions

CIW, QL, CMK, LE, FB, PZ, and WW designed research, and analyzed and interpreted data; CIW, QL, CMK, LE, FB, and PZ performed experiments; DOB, KJ, RE, TWM, THB, and MZ contributed vital reagents; CIW and WW wrote the manuscript, and all authors provided input to the manuscript. All authors read and approved the final manuscript.

## Supplementary Material

Additional file 1: Tables S1 S3 and S4 and Figures S1 to S12. **Table S1.** DNAm profiles for selection of AR-CpGs. **Table S3.** Gene Ontology analysis of the 102 AR-CpG sites. **Table S4.** primers used for pyrosequencing. **Figure S1.** nucleotides and motifs near AR-CpGs. **Figure S2.** enrichment of histone modifications near AR-CpGs. **Figure S3.** DNAm level in age-related hypo- or hypermethylation. **Figure S4.** analysis of AR-CpG sites in an independent dataset. **Figure S5.** age prediction in ESCs and iPSCs. **Figure S6.** flowchart for selection of the epigenetic aging signature. **Figure S7.** DNAm level at CpGs in the neighborhood of five AR-CpG sites. **Figure S8.** gene expression of selected genes with age-related CpG sites. **Figure S9.** DNAm level in different blood subsets. **Figure S10.** influence of blood cell composition on age prediction. **Figure S11.** effect of clinical and lifestyle parameters on age predictions. **Figure S12.** age predictions based on telomere length. Click here for file

Additional file 2: Table S2 Beta values for 102 AR-GpGs from 575 samples. Click here for file
