# Supplementary material for: Aging of blood can be tracked by DNA methylation changes at just three CpG sites
Source: Genome Biol. 2014 Feb 3;15(2):R24. doi: 10.1186/gb-2014-15-2-r24 (PMC4053864; doi:10.1186/gb-2014-15-2-r24)
Supplement: Additional file 1: Tables S1 — S3 and S4 and Figures S1 to S12. Table S1. DNAm profiles for selection of AR-CpGs. Table S3. Gene Ontology analysis of the 102 AR-CpG sites. Table S4. primers used for pyrosequencing. Figure S1. nucleotides and motifs near AR-CpGs. Figure S2. enrichment of histone modifications near AR-CpGs. Figure S3. DNAm level in age-related hypo- or hypermethylation. Figure S4. analysis of AR-CpG sites in an independent dataset. Figure S5. age prediction in ESCs and iPSCs. Figure S6. flowchart for selection of the epigenetic aging signature. Figure S7. DNAm level at CpGs in the neighborhood of five AR-CpG sites. Figure S8. gene expression of selected genes with age-related CpG sites. Figure S9. DNAm level in different blood subsets. Figure S10. influence of blood cell composition on age prediction. Figure S11. effect of clinical and lifestyle parameters on age predictions. Figure S12. age predictions based on telomere length. [file gb-2014-15-2-r24-S1.pdf]

Aging of blood can be tracked by DNA methylation changes at just three CpG sites

Supplemental Material

Carola Ingrid Weidner, Qiong Lin, Carmen Maike Koch, Lewin Eisele, Fabian Beier, Patrick Ziegler, Dirk Olaf Bauerschlag, Karl-Heinz Jöckel, Raimund Erbel, Thomas Walter Mühleisen, Martin Zenke, Tim Henrik Brümmendorf, Wolfgang Wagner

Table S1: DNAm profiles for selection of AR-CpGs.

GEO accession	Ref.	Tissue	Cell type	Samples (n)	Age range (median) in years	Gender (f/m)
GSE19711	[1]	blood	leukocytes	261	52 – 78 (64)	261/0
GSE20242	[2]	blood	$CD14^+$ monocytes	22	16 – 69 (34)	16/6
GSE20242	[2]	blood	$CD4^+$ T cells	18	16 – 69 (29.5)	13/5
GSE23638	[3]	blood	lymphocytes	23	2 – 35 (12)	11/12
GSE20236	[2]	blood	leukocytes	88	49 – 74 (63)	88/0
GSE27317	[4]	cord blood	MNCs	163	0	78/85

All of these datasets were generated on the 27k Illumina BeadChip. Data are accessible under http://www.ncbi.nlm.nih.gov/geo/.

Table S2: beta-values from 102 AR-GpGs of 575 samples.
(provided as separate file)

Table S3: Gene Ontology analysis of the 102 AR-CpG sites.

58 CpG sites with negative correlation (43 genes)

GO category	total genes	changed genes	enrich-ment	log10 (p-value)
GO:0050868_negative_regulation_of_T_cell_activation	39	3	19.9	-3.35
GO:0051250_negative_regulation_of_lymphocyte_activation	51	3	15.2	-3.01
GO:0002695_negative_regulation_of_leukocyte_activation	56	3	13.9	-2.89
GO:0001775_cell_activation	571	8	3.6	-2.87
GO:0050866_negative_regulation_of_cell_activation	62	3	12.5	-2.76
GO:0042752_regulation_of_circadian_rhythm	23	2	22.5	-2.45
GO:0042130_negative_regulation_of_T_cell_proliferation	25	2	20.7	-2.38
GO:0042180_cellular_ketone_metabolic_process	699	8	3.0	-2.32
GO:0002683_negative_regulation_of_immune_system_process	94	3	8.3	-2.25
GO:0070664_negative_regulation_of_leukocyte_proliferation	32	2	16.2	-2.17
GO:0016053_organic_acid_biosynthetic_process	206	4	5.0	-2.10
GO:0046394_carboxylic_acid_biosynthetic_process	206	4	5.0	-2.10

44 CpG sites with positive correlation (32 genes)

GO category	total genes	changed genes	enrich-ment	log10 (p-value)
GO:0070252_actin-mediated_cell_contraction	40	3	22.5	-3.51
GO:0006936_muscle_contraction	209	5	7.2	-3.22
GO:0003012_muscle_system_process	223	5	6.7	-3.10
GO:0019219_regulation_of_nucleobase_nucleoside_nucleotide	2468	17	2.1	-2.94
GO:0051252_regulation_of_RNA_metabolic_process	1598	13	2.5	-2.89
GO:0030326_embryonic_limb_morphogenesis	80	3	11.3	-2.63
GO:0043484_regulation_of_RNA_splicing	22	2	27.3	-2.62
GO:0035108_limb_morphogenesis	91	3	9.9	-2.47
GO:0060173_limb_development	96	3	9.4	-2.41
GO:0048598_embryonic_morphogenesis	325	5	4.6	-2.38
GO:0046058_cAMP_metabolic_process	119	3	7.6	-2.15
GO:0008015_blood_circulation	246	4	4.9	-2.06

Table S4: Primers used for pyrosequencing.

Primer	Sequence
ASPA-for	5´-ATTATTTGGTGAAATGATT-3´
ASPA-rev	5´-CAACCCTATTCTCTAAATCTC-3´
ASPA-seq	5´-CCCTATTCTCTAAATCTCA-3´
ITGA2B-for	5´-TAATTTTTTTTGGGTGATG-3´
ITGA2B-rev	5´-ACCAAAAATAAACAATATACTCAAT-3´
ITGA2B-seq	5´-CAATATACTCAATACTATACCT-3´
PDE4C-for	5´-AGGTTTGTAGTAGGTTGAG-3´
PDE4C-rev	5´-AACTCAAATCCCTCTC-3´
PDE4C-seq	5´-GTTATAGTATGATTAGAGTTT-3´

a

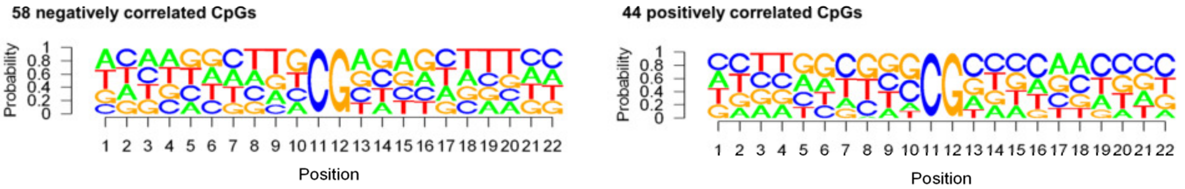

b

58 negatively correlated CpGs

Motif	TF	Frequency	ES	*P*-value
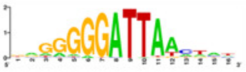	OBOX2	41%	1.89	0.001
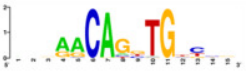	MYF6	74%	1.37	0.001
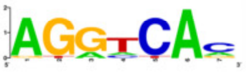	NR4A2	57%	1.50	0.002
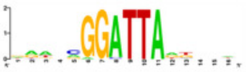	DMBX1	28%	1.87	0.008
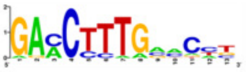	NR2F1	62%	1.35	0.01

44 positively correlated CpGs

Motif	TF	Frequency	ES	*P*-value
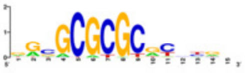	ZFP161	75%	1.45	0.001
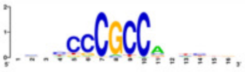	SMAD3	98%	1.18	0.003
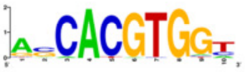	MYC-MAX	48%	1.71	0.004
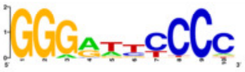	NFKB1	84%	1.30	0.004
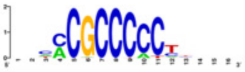	SP4	100%	1.30	0.005

Figure S1: Nucleotides and motifs near AR-CpGs.
(**a**) Frequency of nucleotides next to CpG sites which are either hypomethylated or hypermethylated upon aging. Letter-size indicates the probability of each base at a specific location (AR-CpGs at position 11/12). (**b**) Predicted transcription factor (TF) binding sites within 1 kb up- and down-stream of AR-CpGs. Enrichment score (ES) and p-values are presented for the five most significant results. Significance was calculated by Fisher´s Exact Test.

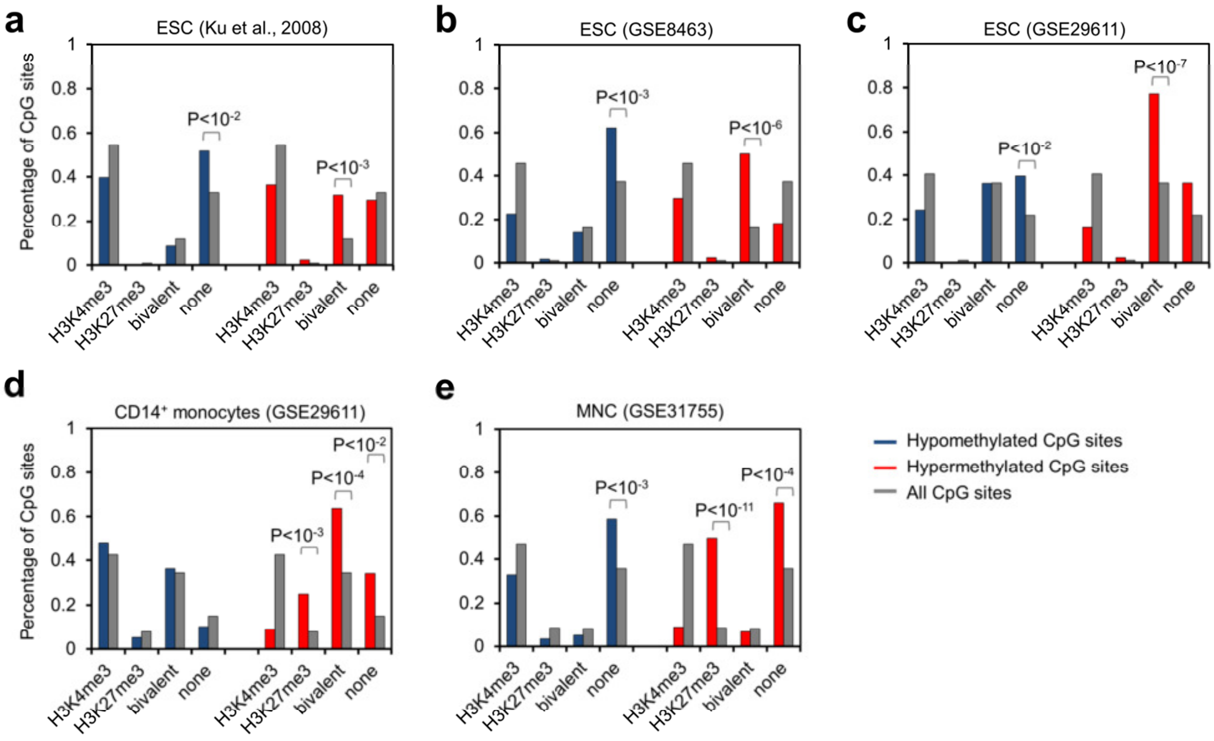

Figure S2: Enrichment of histone modifications near AR-CpGs.
Chromatin immunoprecipitation data for histone modifications (H3K4me3 and H3K27me3) of embryonic stem cells (**a***,* **b** and **c**), $CD14^{+}$ monocytes (**d**), and mononuclear cells (MNCs; **e**) were matched to CpG loci represented on the HumanMethylation27 BeadChip platform. Enrichment of histone modifications in age-related hypomethylation (blue) or AR-hypermethylation (red) was determined in relation to all CpG sites on the array (grey). AR-hypermethylation is significantly enriched in genomic regions which harbor bivalent modifications in ESCs and H3K27me3 modifications in hematopoietic cells. Significance was calculated by Fisher´s Exact Test.

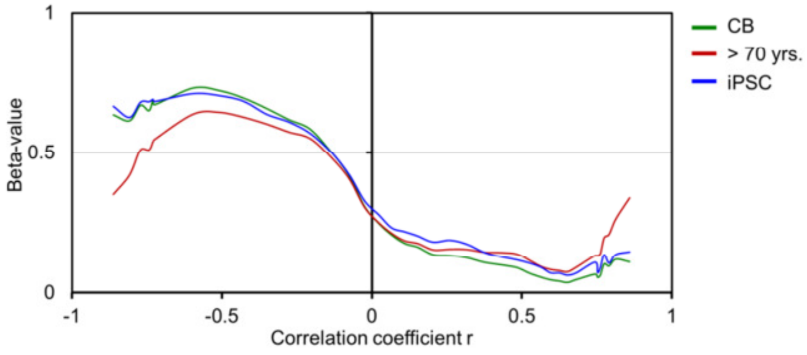

Figure S3: DNAm level in age-related hypo- or hypermethylation.
Pearson correlation was calculated for each of the 27,578 individual CpG sites in relation to age (based on 575 DNAm profiles). The mean DNAm level for CpGs with corresponding r-values (mean of 1,000 CpGs) was plotted for cord blood (CB, green), leucocytes of elderly donors (> 70 years, red), and iPSCs (GSE24676, blue). Overall, CpG sites with AR-hypomethylation are rather highly methylated and *vice versa*. Changes upon aging tend towards a DNAm level of 50%.

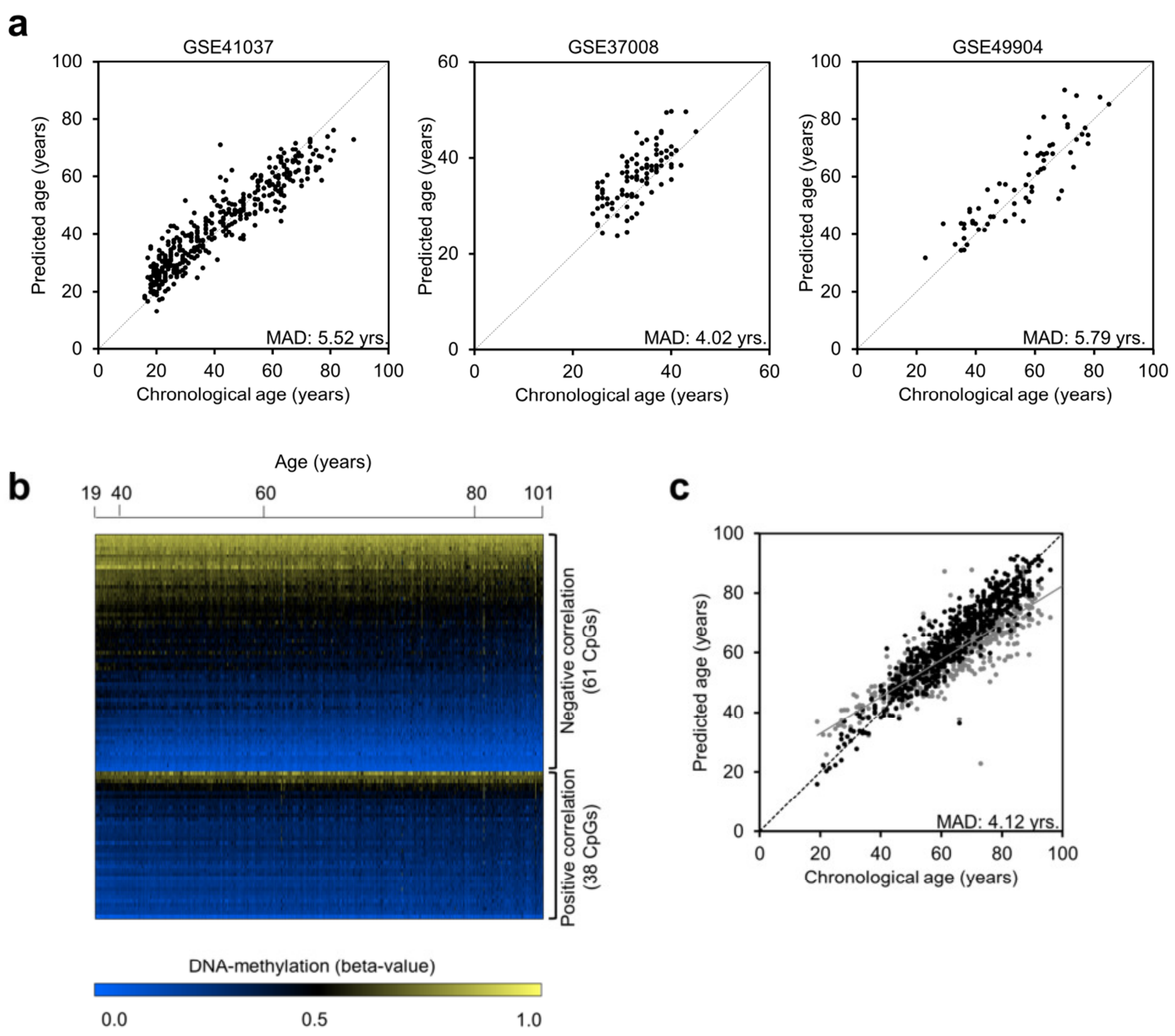

Figure S4: Analysis of AR-CpG sites in independent datasets.

(**a**) Three additional datasets (GSE41037 [5], GSE37008 [6] and GSE49904) [7]) – all based on the HumanMethylation27 BeadChip - were used for further validation of the Epigenetic-Aging-Signature. Age predictions based on 102 CpG sites underlined the precision and reproducibility of the model. (**b**) Additionally, AR-CpG sites were analyzed in the dataset of Hannum and coworkers [8]. This data was generated on the HumanMethylation 450k BeadChip which comprised 99 of our 102 AR-CpGs. Notably, most of the features on this platform are based on a slightly modified DNAm assay (Infinium II beadtype) [9]. (**c**) When we calculated age-predictions based on 99 CpG sites, they clearly correlated with chronological age (grey dots). However, the linear offset indicated a systematic bias which might be due to the three missing CpG sites or to the different assay design of the two microarray platforms. A multivariate model based on the 99 CpGs of the 450k BeadChip facilitated reliable age predictions (MAD: 4.12 years; black) indicating that these CpGs are clearly age-associated. MAD = mean absolute deviation.

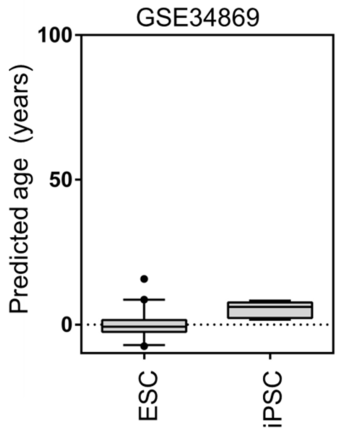

Figure S5: Age prediction in ESCs and iPSCs.
Publically available DNAm profiles of embryonic stem cells (ESCs, n = 19) and induced pluripotent stem cells (iPSCs, n = 5) were used for age prediction applying the multivariate model based on 102 CpG sites (GSE34869) [10].

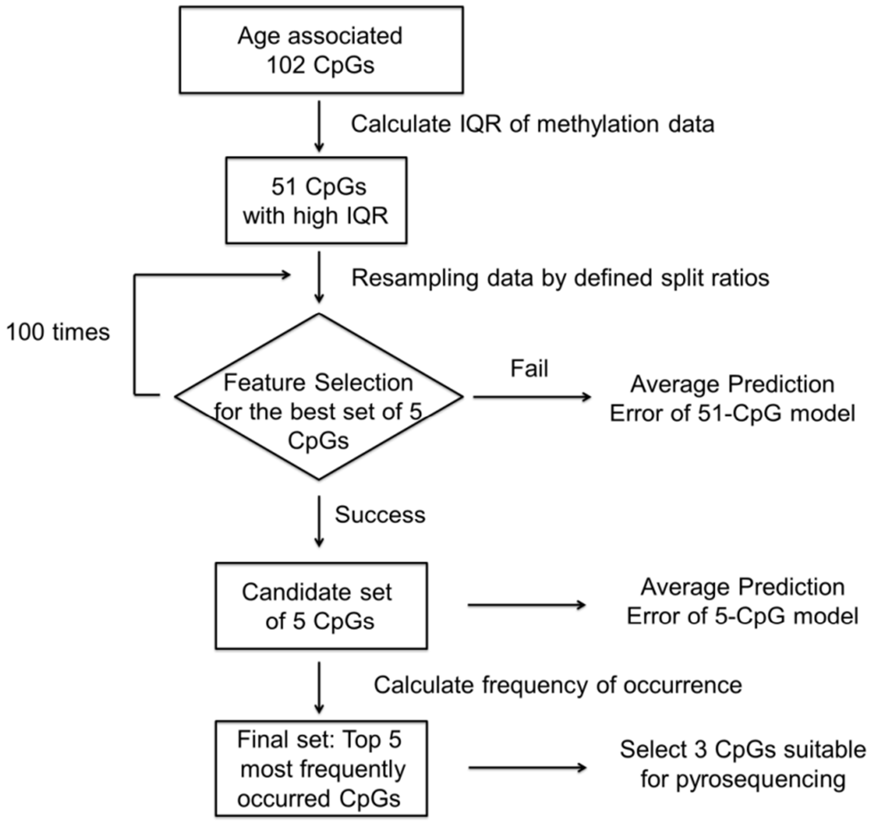

Figure S6: Flowchart for selection of the Epigenetic-Aging-Signature.
Selection of three CpG sites from 102 age-associated pre-filtered loci for pyrosequencing. IQR = interquartile range.

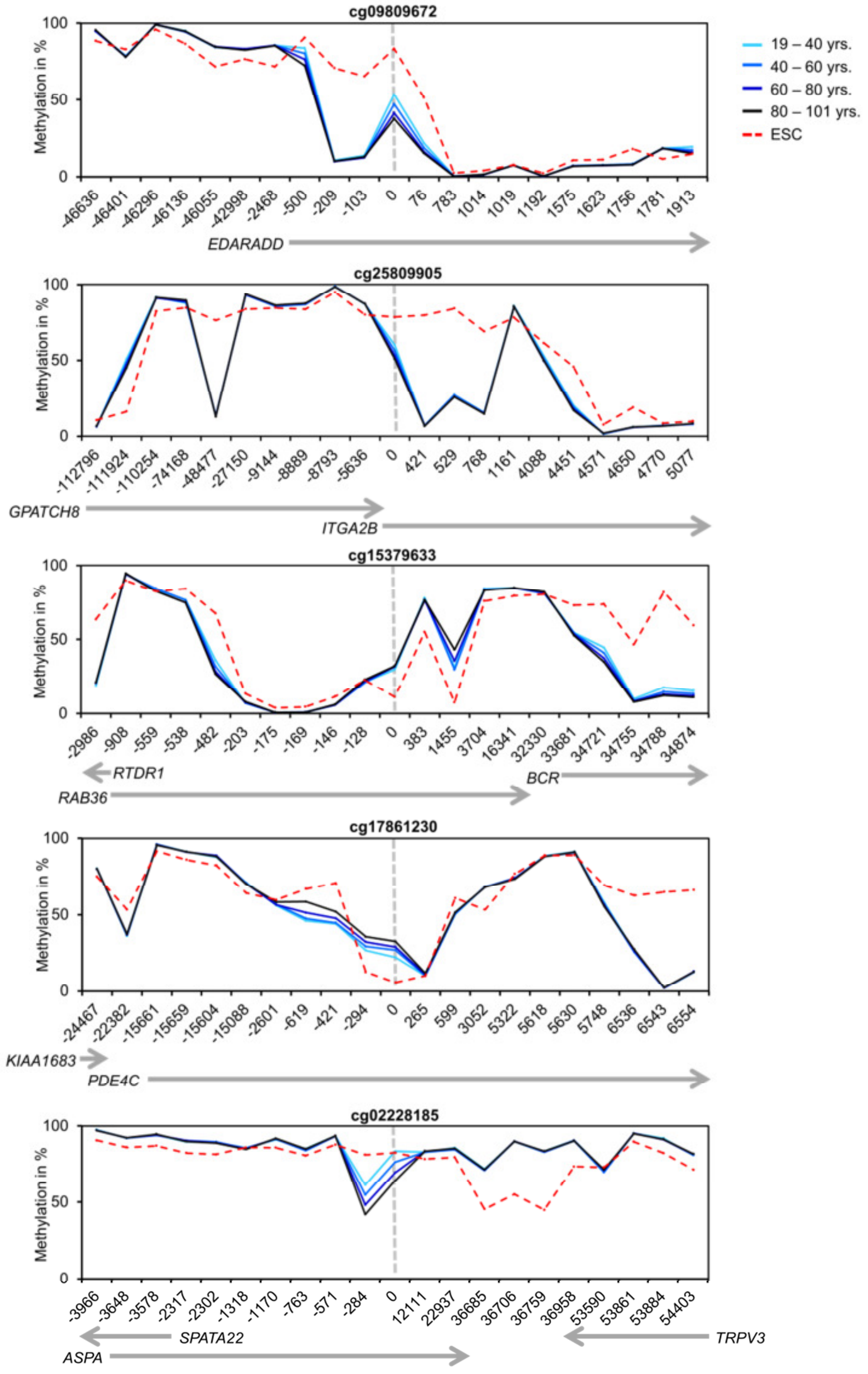

Figure S7: DNAm level at CpGs in the neighborhood of 5 AR-CpG sites.
For the five top AR-CpGs we analyzed DNAm at 10 up- and down-stream located CpG sites on the 450k BeadChip. DNAm was analyzed in blood samples of different age-groups [8] and in relation to embryonic stem cells [11]. The distance to the corresponding AR-CpG site is depicted in base pairs. Grey arrows indicate orientation of the genes. AR-DNAm changes were often also observed in surrounding CpGs but they are not necessarily associated with promoter regions. Notably, the trend of AR-DNAm changes towards younger donors continues in ESCs in each of these genomic regions.

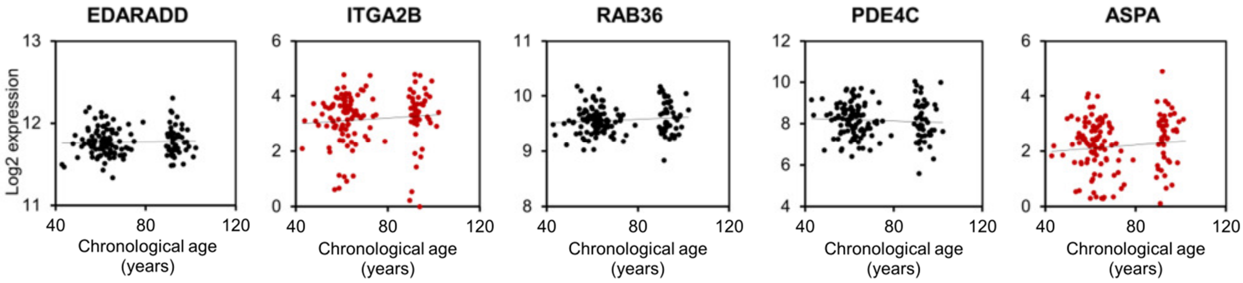

Figure S8: Gene expression of selected genes with age-related CpG sites.
Gene expression profiles of whole blood samples from the Leiden Longevity Study (150 samples; GSE16717) [12] were analyzed for the top 5 AR-CpG loci. *ITGA2B* and *ASPA* were hardly expressed, which is indicated in red. Overall, DNAm changes upon aging were hardly reflected in expression of these genes.

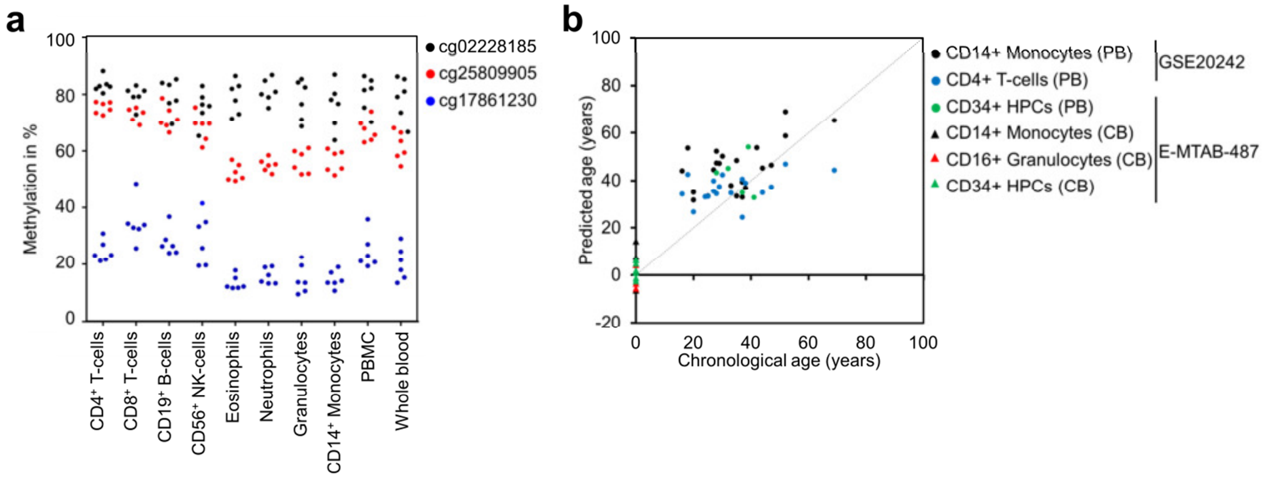

Figure S9: DNAm level in different blood subsets.
Results of the Epigenetic-Aging-Signature might be due to myeloid skewing – a phenomenon particularly observed in the elderly [13]. (**a**) To this end, DNAm profiles of various blood cell types from a dataset of Reinius and coworkers (GSE35069) [14] were analyzed for the three AR-GpG sites of the Epigenetic-Aging-Signature (n=6; age 38±13.6 years). DNAm at cg02228185 depicted a constant level for all blood subsets. Myeloid cells revealed a slightly lower methylation level than lymphoid cells for the CpG sites cg25809905 and cg17861230. As cg17861230 is hypermethylated upon aging (Figure 2d) these results indicate that AR-DNAm is not due to myeloid skewing. (**b**) In addition, age predictions were calculated on available DNAm profiles of different cell types in peripheral blood (PB) and cord blood (CB) (GSE20242 [2] and E-MTAB-487 [15]). For this analysis the multivariate model, based on 3 CpG sites of pyrosequencing data, had to be adopted because the Illumina BeadChips do not cover the neighboring CpG site of cg17861230 (Figure 2c). To this end, we have recalculated the multivariate model based on the initial 575 DNAm profiles (Table S1; GSE20242 has also been included in this training-set but the results are very similar if it would be excluded for this analysis): cg02228185 = α, cg25809905 = β and cg17861230 = γ. The formula for age prediction is: predicted age [in years] = 96.6 – 56.8 α – 64.2 β + 90.5 γ. Overall, age prediction was feasible in all data of isolated hematopoietic subsets that we analyzed – even though the deviation was larger than in pyrosequencing data of whole blood. CB samples were always predicted close to 0. Thus, AR-DNAm changes at the three CpGs cannot only be attributed to changing composition of blood upon aging.

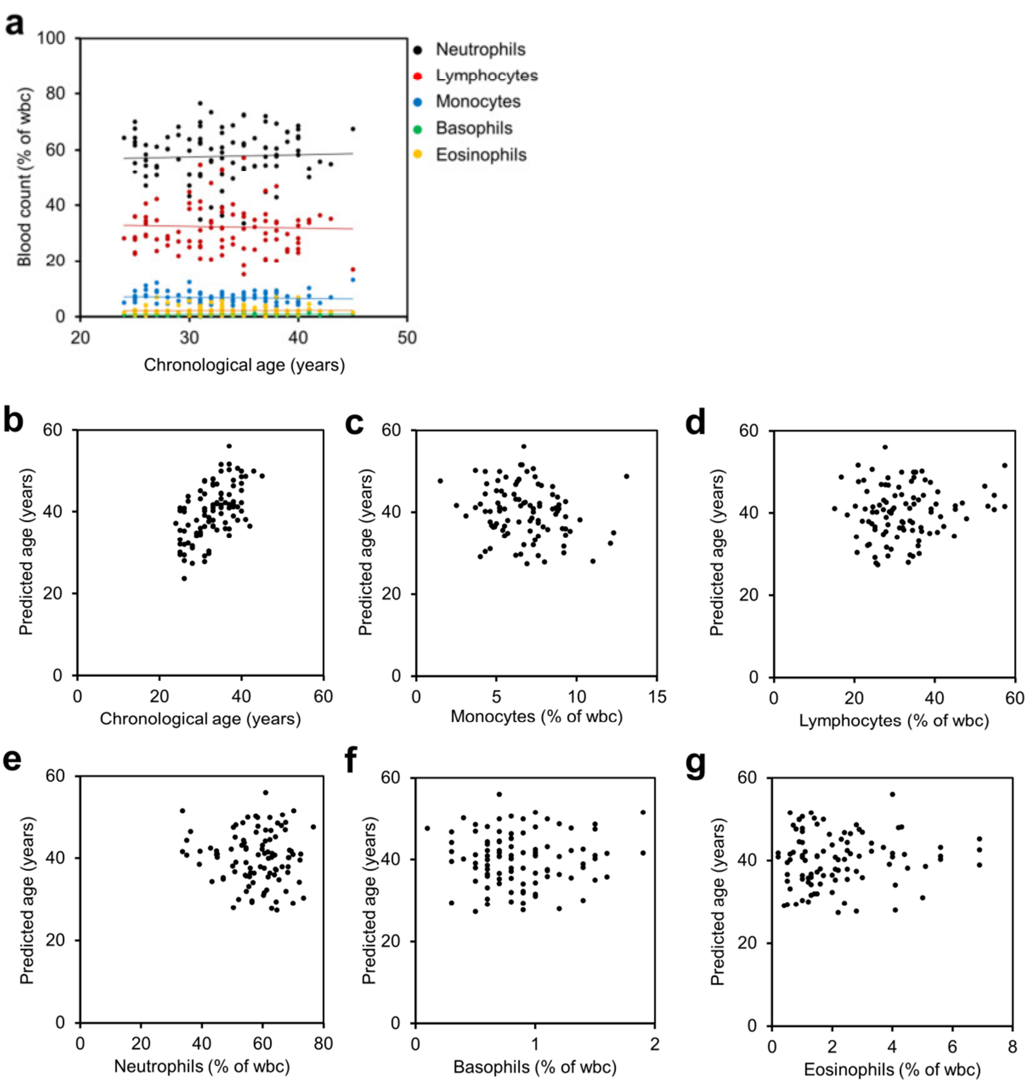

Figure S10: Influence of blood cell composition on age prediction.
Lam and coworkers provided DNAm profiles of 99 blood samples (age 24 - 45) including information on blood counts (GSE37008)[6]. (**a**) In this age range blood counts revealed only very moderate changes upon aging – but there was high variation in the percentage of white blood cells (wbc) between individual samples. (**b**) To further analyze, if the composition of specific cell types has major impact on age-predictions we applied the multivariate model based on 3 CpG sites (multivariate model of Figure S9 for Illumina BeadChip data). Overall, age-predictions were overestimated in this dataset (MAD = 7.57 years) which might be due to technical differences by inter-comparison of different studies. Increased percentages of monocytes (**c**), lymphocytes (**d**), neutrophils (**e**), basophils (**f**), or eosinophils (**g**) did not reveal a systematic effect on age prediction.

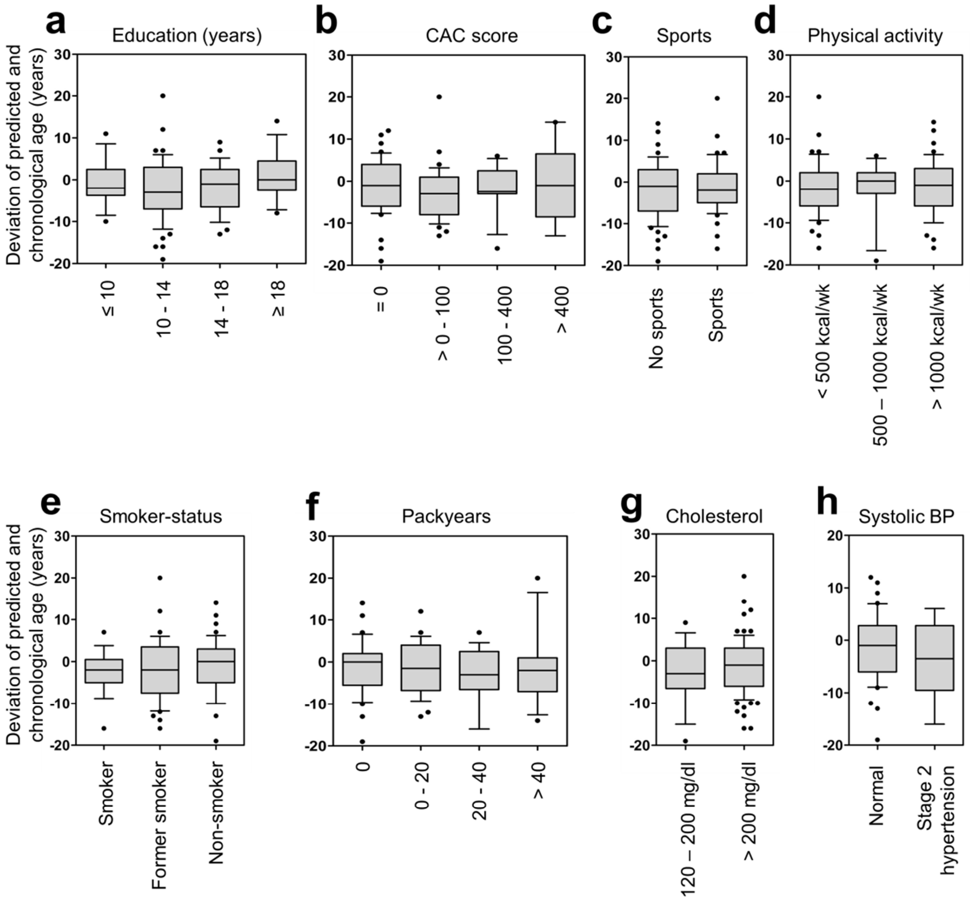

Figure S11: Effect of clinical and lifestyle parameters on age-predictions.
The deviation of predicted and chronological age (residuals) was correlated to the following parameters in 105 samples from the HNR-study: years of education (**a**; *P* = 0.37) [16], coronary-artery calcium (CAC) score (**b**; *P* = 0.79) [17], sports (**c**; no sports or at least once a week; *P* = 0.60) or physical activity (**d**; *P* = 0.62) [18], smoker-status (**e**; *P* = 0.33) and packyears (**f**; *P* = 0.89), cholesterol level (**g**; *P* = 0.82) and systolic blood pressure (**h**; *P* = 0.34) on the age-prediction model. None of the presented parameters was significantly associated with regard to the deviation of predicted and chronological age.

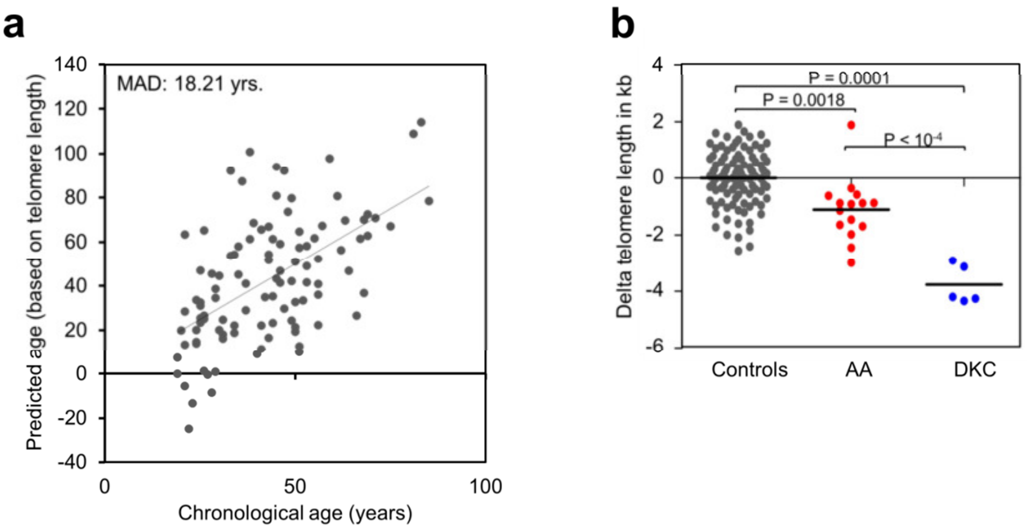

Figure S12: Age-predictions based on telomere length.
(***a***) Age was predicted with regard on telomere length in granulocytes measured by Flow-FISH (MAD 18.21 years). (**b**) Telomere length in relation to age-adjusted mean - given as delta telomere length - is presented for 104 healthy controls, 15 patients with aplastic anemia (AA) and 5 patients with dyskeratosis congenita (DKC). Significance was calculated by two-sided Student´s T-test. MAD = mean absolute deviation of predicted and chronological age.

References for supplemental data

1. Teschendorff AE, Menon U, Gentry-Maharaj A, Ramus SJ, Weisenberger DJ, Shen H, Campan M, Noushmehr H, Bell CG, Maxwell AP, Savage DA, Mueller-Holzner E, Marth C, Kocjan G, Gayther SA, Jones A, Beck S, Wagner W, Laird PW, Jacobs IJ, Widschwendter M: **Age-dependent DNA methylation of genes that are suppressed in stem cells is a hallmark of cancer.** *Genome Res* 2010, **20:** 440-446.

2. Rakyan VK, Down TA, Maslau S, Andrew T, Yang TP, Beyan H, Whittaker P, McCann OT, Finer S, Valdes AM, Leslie RD, Deloukas P, Spector TD: **Human aging-associated DNA hypermethylation occurs preferentially at bivalent chromatin domains.** *Genome Res* 2010, **20:** 434-439.

3. Chen YA, Choufani S, Ferreira JC, Grafodatskaya D, Butcher DT, Weksberg R: **Sequence overlap between autosomal and sex-linked probes on the Illumina HumanMethylation27 microarray.** *Genomics* 2011, **97:** 214-222.

4. Adkins RM, Thomas F, Tylavsky FA, Krushkal J: **Parental ages and levels of DNA methylation in the newborn are correlated.** *BMC Med Genet* 2011, **12:** 47.

5. Horvath S, Zhang Y, Langfelder P, Kahn RS, Boks MP, van EK, van den Berg LH, Ophoff RA: **Aging effects on DNA methylation modules in human brain and blood tissue.** *Genome Biol* 2012, **13:** R97.

6. Lam LL, Emberly E, Fraser HB, Neumann SM, Chen E, Miller GE, Kobor MS: **Factors underlying variable DNA methylation in a human community cohort.** *Proc Natl Acad Sci U S A* 2012, **109 Suppl 2:** 17253-17260.

7. Day K, Waite LL, Thalacker-Mercer A, West A, Bamman MM, Brooks JD, Myers RM, Absher D: **Differential DNA methylation with age displays both common and dynamic features across human tissues that are influenced by CpG landscape.** *Genome Biol* 2013, **14:** R102.

8. Hannum G, Guinney J, Zhao L, Zhang L, Hughes G, Sadda S, Klotzle B, Bibikova M, Fan JB, Gao Y, Deconde R, Chen M, Rajapakse I, Friend S, Ideker T, Zhang K: **Genome-wide Methylation Profiles Reveal Quantitative Views of Human Aging Rates.** *Mol Cell* 2013, **49:** 459-367.

9. Bibikova M, Barnes B, Tsan C, Ho V, Klotzle B, Le JM, Delano D, Zhang L, Schroth GP, Gunderson KL, Fan JB, Shen R: **High density DNA methylation array with single CpG site resolution.** *Genomics* 2011, **98:** 288-295.

10. Mallon BS, Chenoweth JG, Johnson KR, Hamilton RS, Tesar PJ, Yavatkar AS, Tyson LJ, Park K, Chen KG, Fann YC, McKay RD: **StemCellDB: the human pluripotent stem cell database at the National Institutes of Health.** *Stem Cell Res* 2013, **10:** 57-66.

11. Shao K, Koch CM, Gupta MK, Lin Q, Lenz M, Laufs S, Denecke B, Schmidt M, Linke M, Hennies HC, Hscheler J, Zenke M, Zechner U, Saric T, Wagner W: **Induced Pluripotent Mesenchymal Stromal Cell Clones Retain Donor-Derived Differences in DNA Methylation Profiles.** *Molecular Therapy* 2013, **21:** 240-250.

12. Passtoors WM, Boer JM, Goeman JJ, Akker EB, Deelen J, Zwaan BJ, Scarborough A, Breggen R, Vossen RH, Houwing-Duistermaat JJ, Ommen GJ, Westendorp RG, Heemst D, Craen AJ, White AJ, Gunn DA, Beekman M, Slagboom PE: **Transcriptional profiling of human familial longevity indicates a role for ASF1A and IL7R.** *PLoS ONE* 2012, **7:** e27759.

13. Pang WW, Price EA, Sahoo D, Beerman I, Maloney WJ, Rossi DJ, Schrier SL, Weissman IL: **Human bone marrow hematopoietic stem cells are increased in frequency and myeloid-biased with age.** *Proc Natl Acad Sci U S A* 2011, **108:** 20012-20017.

14. Reinius LE, Acevedo N, Joerink M, Pershagen G, Dahlen SE, Greco D, Soderhall C, Scheynius A, Kere J: **Differential DNA methylation in purified human blood cells: implications for cell lineage and studies on disease susceptibility.** *PLoS ONE* 2012, **7:** e41361.

15. Bocker MT, Hellwig I, Breiling A, Eckstein V, Ho AD, Lyko F: **Genome-wide promoter DNA methylation dynamics of human hematopoietic progenitor cells during differentiation and aging.** *Blood* 2011, **117:** e182-e189.

16. Weyers S, Dragano N, Möbus S, Beck EM, Stang A, Möhlenkamp S, Jöckel KH, Erbel R, Siegrist J: **Low socio-economic position is associated with poor social networks and social support: results from the Heinz Nixdorf Recall Study.** *Int J Equity Health* 2008, **7:** 13.

17. Erbel R, Möhlenkamp S, Möbus S, Schmermund A, Lehmann N, Stang A, Dragano N, Grönemeyer D, Seibel R, Kälsch H, Bröcker-Preuss M, Mann K, Siegrist J, Jöckel KH: **Coronary risk stratification, discrimination, and reclassification improvement based on quantification of subclinical coronary atherosclerosis: the Heinz Nixdorf Recall study.** *J Am Coll Cardiol* 2010, **56:** 1397-1406.

18. Sesso HD, Paffenbarger RS, Ha T, Lee IM: **Physical activity and cardiovascular disease risk in middle-aged and older women.** *Am J Epidemiol* 1999, **150:** 408-416.
